# miRNAs as Modern Biomarkers in Asthma Therapy

**DOI:** 10.3390/ijms241411499

**Published:** 2023-07-15

**Authors:** Natalia Kierbiedź-Guzik, Barbara Sozańska

**Affiliations:** 1 14th Paediatric Ward—Pulmonology and Allergology, J. Gromkowski Provincial Specialist Hospital, ul. Koszarowa 5, 51-149 Wrocław, Poland; 2 1st Department and Clinic of Paediatrics, Allergology and Cardiology Wrocław Medical University, ul. Chałubińskiego 2a, 50-368 Wrocław, Poland; barbara.sozanska@umw.edu.pl

**Keywords:** asthma, miRNA, antagomir, biomarkers, response, treatment, glucocorticosteroids, beta-mimetic, biological therapy, pathways, infection

## Abstract

Asthma is a chronic inflammatory disease of the airways characterized by shortness of breath, chest tightness, coughing, and wheezing. For several decades (approximately 30 years), miRNAs and their role in asthma have been of constant interest among scientists. These small, non-coding RNA fragments, 18–25 nucleotides long, regulate gene expression at the post-transcriptional level by binding to the target mRNA. In this way, they affect several biological processes, e.g., shaping airway structures, producing cytokines and immune mediators, and controlling defense mechanisms. Publications confirm their potential role in the diagnosis and monitoring of the disease, but only some articles address the use of miRNAs in the treatment of asthma. The following paper reviews the latest available studies and presents miRNAs as a useful tool for predicting the effectiveness of the included treatment, early diagnosis of exacerbations, and in assessing patient compliance for different groups of drugs used in asthma. The latest known pathways underlying the pathogenesis of the disease, which are associated with a change in miRNA expression, may be precise targets of therapeutic activity in the future.

## 1. Introduction

**Figure 1:**
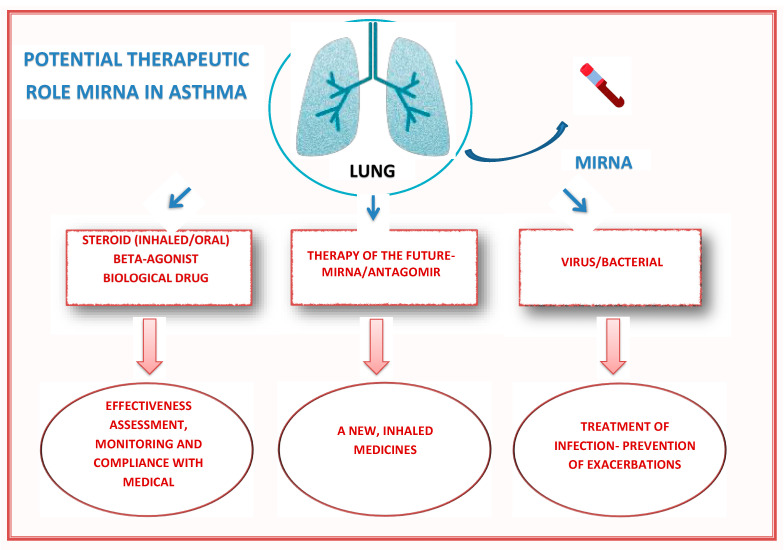
miRNAs and their potential use in asthma.

Asthma is a chronic inflammatory disease of the airways characterized by shortness of breath, chest tightness, wheezing, and coughing, affecting nearly 300 million people worldwide, and an additional 100 million people are expected to be affected by the end of 2025 [1]. Environmental factors and genetic conditions predispose the development of the disease, and their exact pathomechanism is not known [2]. A particular clinical challenge is severe asthma, which, despite the use of high doses of inhaled corticosteroids (ICS) and a second controller and/or oral corticosteroids, still cannot be adequately controlled [3]. In adults, 5–10% are affected by this disease [4], and the exact statistics among children are unknown [5]. This problem is estimated to affect about 5% of the pediatric population [6]. In addition, its various phenotypes can be distinguished, considering the cells involved in the systemic inflammatory process (eosinophilic, neutrophilic, and mixed form) [7,8]. In most cases, severe asthma is characterized by atopy, higher levels of IgE and eosinophils in the blood, increased levels of FeNO (fractional exhaled nitric oxide) in exhaled air, and, usually, positive skin tests after contact with an allergen. Neutrophilic asthma is characterized by the predominance of neutrophils in the blood and sputum and the involvement of Th1 (T helper type 1) and Th17 (T helper type 17) cells in the inflammatory process [9]. It has been proven that Th17 lymphocytes increase neutrophil infiltration, which may cause increased inflammation in the airways and contribute to severe cases of steroid-resistant disease [10,11]. The phenotype of severe asthma is very complex and understanding the molecular basis of its course will allow for greater control of disease symptoms and an improved response to treatment. The hope for understanding these biological pathways is provided by miRNA molecules, which have been an intense subject of scientific interest in recent years. Their broad impact on the processes involved in the pathogenesis of asthma has been confirmed (including the proliferation of smooth muscle cells, increased mucus production, collagen, and overactivity of the respiratory tract) [12]. These are small, non-coding RNA biomolecules, 18–25 nucleotides long, and regulate gene expression at the post-transcriptional level by binding to the target mRNA (messenger RNA) [13]. Due to their role in multiple cellular processes, such as inflammatory and immunomodulatory processes, they are an important future target of therapeutic activities leading to increased and improved sensitivity to steroid therapy [14]. The following paper summarizes the results of recent studies showing miRNAs and their role as molecules in monitoring the effectiveness of therapy, assessing the response to the introduced treatment, and examples of their experimental use in asthma therapy. The paper pays special attention to studies conducted among the pediatric population. However, due to their small number, the review also included studies conducted on groups of adult patients. The potential use of miRNAs in the treatment of asthma is summarized and presented in Figure 1 below.

## 2. miRNAs and Individual Groups of Drugs Used in Asthma (Glucocorticosteroids, Short-Acting Beta-Agonists, and Biological Therapy)

Currently, many publications show miRNAs as potential asthma diagnostic biomarkers [13,15,16,17]. Few studies have also drawn attention to the relationship between miRNAs and a particular therapeutic regimen. The following chapter divides the studies according to the main groups of medicines used in asthma and shows their relationship with individual miRNAs, thus confirming their use as predictive biomarkers, and in monitoring and evaluating the effectiveness of the treatment. 

The first object of consideration will be inhaled glucocorticosteroids (ICS), the primary asthma drug [18]. They soothe the inflammatory process in the respiratory tract and prevent future disease exacerbations. Their mechanism of action is complex and can be divided into genomic and non-genomic. The first directly relates to the effect on gene transcription, causing their transactivation (increased transcription) or transrepression (reduced transcription). The other activates signaling pathways of kinases, G proteins, and ion channels. Their anti-inflammatory effect associated with the inhibition of cytokine production is used in clinical practice [19]. A diverse response to ICS among patients is constantly observed. A relationship between the response and the change in the concentration of individual miRNA molecules in the blood and their direct impact on given biological processes was noted. Among the pediatric population aged 9.0 ± 2.1 years who suffer from asthma and were using inhaled glucocorticosteroids for 4 years, it was shown that two biomolecules—miRNA-155-5p and miRNA-532-5p—can be used to predict the response to the applied treatment. These miRNAs affect the transcription of NF-κB in airway epithelial cells (nuclear factor kappa-light-chain-enhancer of activated B cells), the expression of which is reduced (transrepression) by dexamethasone. The transcription factor NF-κB regulates many aspects of innate and adaptive immune function and is a crucial mediator of the inflammatory response. Consequently, its excessive activation leads to the initiation of pathological processes of many inflammatory diseases, including asthma [20]. miRNA-155-5p inhibits NF-κB transrepression, while miRNA-532-5p increases it. Interestingly, with the increase in the concentration of miRNA155-5p in the blood, the FEV1% (forced expiratory volume during the first second of expiration) improves, and patients have a better response to ICS, while the opposite effect was noted for miRNA-532-5p [21]. Why is it that miRNA-155-5p causes a better therapeutic response even though it intensifies the synthesis of the pro-inflammatory factor NF-κB? The results of other publications may provide a solution to this problem. It has been shown that the expression of miRNA-155-5p is predominantly in the serum in healthy individuals [22], and in asthmatics, it is in the bronchial epithelial cells [23]. Therefore, it can be assumed that the concentration of this molecule is increased in the blood of patients with a milder form of the disease, which is characterized by better sensitivity to ICS. Moreover, miRNA-155, in addition to predicting the effectiveness of the steroids used, can be used as a future therapeutic target in asthma (chapter: miRNA and new directions in asthma treatment). The above paragraph provides examples of miRNA molecules (miRNA-155-5p and miRNA 532-5p) that may be biomarkers for assessing the response to ICS in pediatric asthma.

Another molecule tested in the pediatric population was miRNA-21. It has been shown that miRNA-21 can be successfully used as a non-invasive diagnostic marker of asthma, showing a positive correlation with blood and sputum eosinophilia and IL-4, and a negative correlation with the FEV1 and IL-12 concentration [24]. Among the pediatric group aged 9.0 ± 2.7 years, its expression was increased in children not receiving ICS and in steroid-resistant cases of the disease (SR). In the above study, serum miRNA-21 has a high therapeutic predictive value in differentiating patients with ICS-sensitive and ICS-resistant asthma. It additionally reduces the risk of side effects and spares patients the disappointment of treatment failure. NF-κB and activator protein 1 (AP-1) show affinity for the promoter of the miRNA-21 gene, enhancing its synthesis. Using inhaled steroids reduces the expression of the above transcription factors, explaining the decrease in the serum miRNA-21 concentration [25]. In another project conducted on *mice*, miRNA-21 has been shown to play an essential role in developing severe, steroid-resistant asthma. It is well-known that infections are one of the leading causes of exacerbation in chronic inflammatory diseases of the respiratory tract. An infection leads to an increase in miRNA-21, the molecular goal of which is to inhibit *PTEN* (phosphatase and tensin homologue), belonging to the group of suppressor genes, which results in the activation of one of the isoforms of phosphatidylinositol 3-kinase (PI3K), which leads to the phosphorylation and reduction of HDCA2 (histone deacetylate 2) levels. The miRNA-21/*PTEN*/PI3K/HDAC2 pathway is responsible for developing severe, treatment-resistant disease. Experimental use of the intranasal form of miRNA-21, anti-miRNA-21, and PI3K inhibitor leads to a similar therapeutic effect as the use of steroids in asthma without signs of infection [26]. In 160 children aged 5–12 years using ICS for a year and selected from the CAMP (Childhood Asthma Management Program) group, miRNA-146b, miRNA-206, and miRNA-720 together with the patient’s clinical characteristics helped to identify episodes of exacerbations, and in assessing the effectiveness and the need to escalate therapy. This will allow for the selection of people who are more likely to respond to steroids, minimizing the risk of disease complications, and reducing the cost of medical services. The relationship between the above molecules and the pathways responsible for the development of asthma has been proven [27]. 

The paragraphs mentioned above highlight studies where children were the focus. The following sections focus on adult patients and an induced experimental model of asthma in *mice*.

Another molecule associated with steroid treatment is miRNA-144-3p. In adult patients with severe disease requiring high daily doses of inhaled ICS, oral steroids, or leukotriene receptor antagonist drugs, higher levels of this molecule in the lung tissue have been shown. Its level correlates with worse lung function, eosinophilia, and type 2 inflammatory cytokines. miRNA-144-3p is responsible for the increased expression of genes whose protein products lead to the development of asthma. A single biomarker in conjunction with the clinical characteristics may be helpful in the selection of patients who are more likely to require therapy based on biological drugs [28,29]. miRNA-146a is an important modulator of the inflammatory process; it inhibits the activation of NF-κB via the TLR (toll-like receptor) and reduces the deposition of mucoproteins that contribute to airway remodeling in asthma [30]. Increased levels of this molecule have been reported in the airway epithelial cells and serum of adult patients, necessitating higher doses of inhaled steroids. ICS caused a decrease in the expression of NF-κB and miRNA-146a, while the exogenous introduction of this molecule enhanced the anti-inflammatory effect of the steroids. Increased inflammation induces the expression of miRNA-146a, the primary task of which is its reduction (feedback mechanism). Monitoring the level of this biomarker can help predict the response to glucocorticosteroids, and its synthetic equivalent can be used therapeutically to overcome steroid resistance in patients with severe asthma [31]. In another study by Swedish scientists among adults diagnosed with mild and moderate asthma, the increased expression of miRNA-155 and -146 was found in the blood serum, which additionally increased after the introduction of ICS therapy. Since these medications mainly suppress the type 2 immune response, they may increase the synthesis of post-transcriptional regulators such as miRNA-155 and -146. These biomolecules alleviate asthma symptoms by inhibiting the cells involved in the type 2 response. In addition, common molecular target genes responsible for leukocyte regulation and the response to glucocorticosteroids have been demonstrated for these molecules [32].

In addition, miRNAs can affect the translation of the target protein of the steroid receptor (GR), making steroid treatment ineffective and leading to the development of a resistant form of the disease [33]. One of these molecules is miRNA-9, which is increased upon exposure to IFN (interferon) and LPS (lipopolysaccharide) in macrophages isolated from *mice* lung tissue. The molecular mechanism of steroid resistance is complex. There is a decrease in PP2A (protein phosphatase 2A) activity, which leads to the activation of JNK1 (c-Jun N-terminal kinase), which is responsible for increased steroid receptor phosphorylation. The consequence of these events is impaired translocation to the cell nucleus, leading to ineffectiveness of the steroid therapy. This process is reversed using antagomir (anti-miRNA-9), which restores the PP2A function [34]. A similar relationship to that of the miRNA-9 molecule was shown for miRNA-433 [35] and miRNA-18a [36]. In studies conducted on *mice*, the increase in these biomolecules inhibited GR translocation into the cell nucleus.

Inhaled glucocorticosteroids continue to be the basic drugs controlling the course of asthma. Patients with a severe form of the disease are forced to use them in high doses, often with or without additional treatment, to reduce their activity. Oral corticosteroids (OCS) are drugs reserved for such cases, e.g., uncontrolled asthma and its exacerbations. The need for their long-term use is associated with the occurrence of side effects (e.g., obesity/overweight, gastrointestinal bleeding, hirsutism, steroid acne, muscle mass reduction, psychiatric disorders) and the development of other diseases (such as diabetes, cataracts, osteoporosis, hypertension, iatrogenic Cushing’s syndrome) [37,38]. A study conducted on a group of adult patients with severe asthma who used OCS or did not take the drug showed a difference in the expression of 11 miRNA molecules between the mentioned groups (increased their concentration in the serum of subjects receiving oral steroids). The analysis was performed using Next Generation Sequencing (NGS). Then, after validation by RT-qPCR, it was confirmed that five biomolecules (miRNA-148b-3p, -221-5p, -618, -941, and -769-5p) in blood serum and three in lung tissue samples (miRNA-144-3p, -144-5p, and -451a) showed an increased concentration among OCS recipients. In the multivariate logistic regression model, the combination of miRNA-221-5p and -miRNA-769-5p in the serum was the best for differentiating severe asthma patients treated and untreated with OCS. The participation of these molecules in the pathways responsible for the development of asthma has been confirmed. They were responsible for the changed expression of FOXO3 (forkhead box O3a), PTEN, and MAPK3 (mitogen-activated protein kinase 3), causing, among others, an increased concentration of IgE and excessive synthesis of pro-inflammatory cytokines [39,40]. This is one of the first publications showing the relationship between miRNAs and oral steroids. The obtained results of the above study help differentiate people receiving and not taking OCS. This may be practical in assessing the compliance in treated patients who do not respond to the prescribed oral steroid therapy [41].

As a brief summary of the results of the above publications combining miRNAs with steroid treatment, their potential, huge role in predicting effectiveness, assessing the risk of exacerbations, and observing patient compliance with medical recommendations has been confirmed. They have a broad impact not only on the biological processes responsible for the development of asthma, but also on the mechanism of action of glucocorticosteroids (as described earlier, the effect on the expression of the steroid receptor protein); thus, they are of great importance in the final therapeutic effect.

β2-agonists (SABAs, short acting beta agonists) are another group of drugs that are used in the temporary relief of severe asthma symptoms [42]. Regardless of the severity of the disease and often despite treatment, patients can experience episodes of acute exacerbations and loss of control of the disease, which is where these preparations are required. Their mechanism of action is based on inhibiting the contraction and then promoting relaxation of the smooth muscles of the respiratory tract through the activation of beta-2 adrenergic receptors (ADRB2), and the increased production of cyclic adenosine monophosphoric acid is responsible for this [43]. A study in adult asthmatics who were sensitive and unresponsive to salmeterol, an example of a β2-agonist, showed higher serum miRNA-16 expression levels in drug-resistant patients. This molecule leads to a decrease in ADRB2 expression and, consequently, results in lower FEV1 values, which confirms the positive correlation between the level of the β2-agonist receptor and the lung function index. The results of the above publication show miRNA-16 as a potential biomarker for assessing the effectiveness of therapy [44]. Another molecule proven to be interestingly related to the given group of drugs is miRNA-let-7f. Airway smooth muscle cells were collected from healthy individuals and asthmatics. They were then cultured in a laboratory setting and simultaneously exposed to the β-agonist for 18 h. An increase in the concentration of miRNA-let-7f was observed only for cells derived from asthmatics, which resulted in the inhibition of ADRB2 translation (decrease in the protein product by nearly 90%). CREB (cAMP receptor element-binding protein) is involved in this process, where its activation leads to binding to the promoter of the miRNA-let-7f gene, which in turn results in the increased production of this molecule. The reversal of the process described above was obtained by inhibiting CREB and the miRNA. Interestingly, this phenomenon has been described only in the case of cells taken from sick people and illustrates one of the possible mechanisms of tachyphylaxis among β2-agonists. This confirms its key importance in the pathogenesis of the disease and in the ineffectiveness of treatment [45].

The cited results of studies show an interesting relationship between miRNAs and ad hoc preparations used in asthma exacerbations. Due to the small number of publications on this subject, this is a further interesting research direction.

In recent years, biological drugs available on the market have been mainly used in severe and uncontrolled asthma among the pediatric population. They alleviate the symptoms of the disease, improve lung function, reduce the need for oral glucocorticosteroids, and optimize patients’ quality of life [46]. The mechanism of their action is based on the inhibition of the inflammatory response with the involvement of Th2 (T helper 2 cells), IgE immunoglobulins, and IL-4, 5, and 13 [47]. After showing many interesting relationships between miRNAs and drugs used in asthma, such an attempt was made for modern biological preparations. In a group of adult patients with severe eosinophilic asthma, mepolizumab and reslizumab were included in the treatment. These are humanized immunoglobulin G (IgG) monoclonal antibodies with a high affinity for IL-5, neutralizing this cytokine by binding to epitopes on the IL-5-Rα binding domain. At 8 weeks from the start of the project, an increase in the expression of miRNA-338-3p was noted in the subjects. In this study, the relationship of this molecule with the regulation of the expression of genes involved in the development of asthma, including *MAPK* and *TGF-β* (transforming growth factor beta 1), was confirmed by bioinformatics analysis. They are related to the remodeling of the respiratory tract. Thus, miRNA-338-3p may become a new, useful biomarker of the early response to mepolizumab and reslizumab [48], like L-selectin and KL-6 molecules (Krebs von den lungen-6). Their potential role as new markers of the early response only to mepolizumab was confirmed [49]. Benralizumab is a monoclonal antibody that binds to the alpha subunit of the IL-5 receptor and significantly reduces the number of eosinophils in the peripheral blood. In addition, after 8 weeks of therapy, a change in the concentration of three molecules—miRNA-1246, miRNA-5100, and miRNA-338-3p—was observed in adults diagnosed with severe eosinophilic asthma. The detailed analysis showed that they are regulators of the *MAPK* signaling pathway, which controls crucial genes involved in the pathogenesis of asthma. By monitoring the levels of these miRNAs after the first dose of benralizumab, we can easily assess whether the mechanisms responsible for controlling asthma in these patients have been restored and, subsequently, if their health has improved [50]. An interesting relationship was demonstrated in the group of adults receiving benralizumab at the beginning and in experimental week 4, 8, 16, and 24. This drug has been found to affect the miRNA-21/*PTEN*/PI3K/HDAC2 pathway, which is involved in the development of steroid-resistant asthma, as mentioned in earlier sections of the paper. There was a significant decrease in miRNA-21-5p, which subsequently inactivated PI3K/AKT, ultimately leading to an increase in the expression of genes related to the steroid response, including the glucocorticosteroid receptor. The study provides interesting data where using a biological drug increases the chances of restoring the effectiveness of steroid therapy in patients with severe asthma [51]. Dupilumab is a monoclonal antibody that blocks the receptor for IL-4 and IL-13 and is also approved for treating severe asthma with a predominance for type 2 inflammation. This medicine was used in four subjects with a confirmed diagnosis of chronic sinusitis with nasal polyps (without a diagnosis of asthma). At the end of the treatment, a decrease in the expression of miRNAs-25-3p and -185-5p was shown in the blood. As was proven, they are involved in the inflammatory process, T lymphocyte regulation, and drug resistance, thanks to which we note a significant clinical improvement among the subjects after using the drug [52]. Currently, no studies show the relationship between Dupilumab and miRNAs in asthmatics. It is a promising future research direction.

## 3. miRNAs and Pathways Responsible for the Development of Asthma: The Future Direction of Therapeutic Action

**Figure 2:**
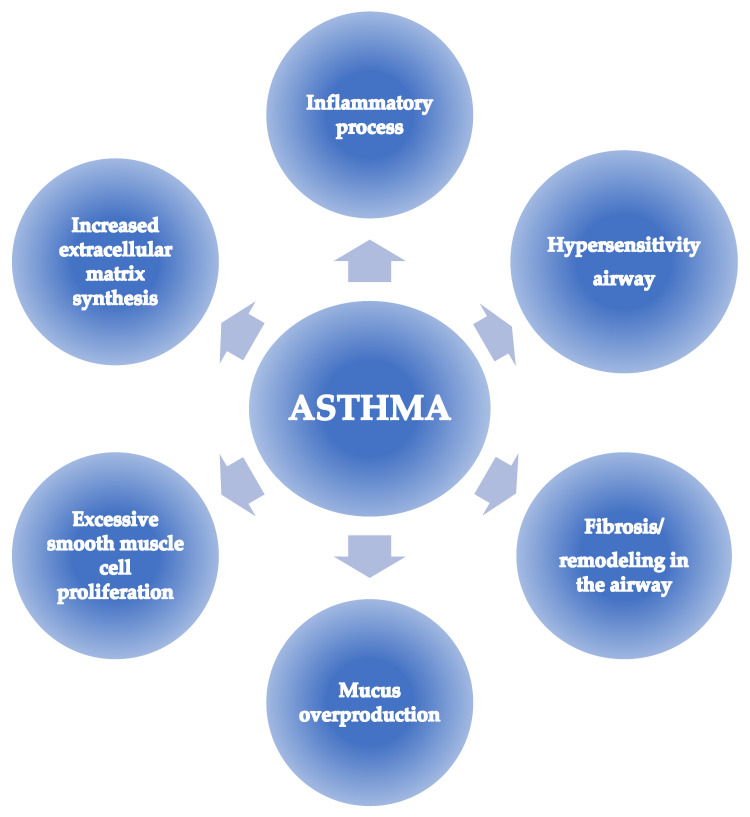
Target direction of therapeutic actions in asthma.

The use of synthetic miRNAs and antagomirs (anti-miRNAs) to inhibit the pathways responsible for the pathogenesis of asthma is a promising target for future therapeutic activities. Anti-miRNA molecules are small interfering oligonucleotides that bind to the target miRNA and prevent them from being processed by the RNA-induced silencing complex (RISC). miRNA mimetics are made of two strands and restore the function of their endogenous counterpart [53]. Below, the results of some of the latest publications revealing yet unknown pathways involved in the development of asthma are presented. This chapter focuses on potential translational research in *mice*, as previous sections have focused on experiments and discoveries in humans. Figure 2 below shows the processes in the airways of people with asthma, a potential target for therapeutic actions.

In addition to the prognostic role of miRNA-155-5p in assessing the response to treatment, this molecule may become an object of therapeutic action in the future. In an experimental study conducted on female *mice*, an increase in the concentration of the miRNA-155 molecule in the lung tissue of animals was noted after inducing asthma by the supply of ovalbumin. It was responsible for the increased synthesis of interleukins 4, 5, and 13 in the bronchoalveolar lavage fluid and the hyperreactivity of the airways. The inflammatory process was reduced when the anti-miRNA-155 counterpart was delivered to the airways with a lentiviral vector. In this way, the miRNA-155 molecule may become a new target for treating allergic inflammatory diseases [54]. In laboratory conditions, asthma was induced in *mice* by exposure to ovalbumin, and the resulting miRNA profile was then analyzed. It has been shown that miRNA-3162-3p is associated with the increased production of active beta-catenin, intensifying the inflammatory process and hyperreactivity of the airways. The use of anti-miRNA-3162-3p was compared in the use of dexamethasone and resulted in the inhibition of the Wnt/beta-catenin pathway [55,56]. Similar results between the target Wnt/beta-catenin pathway and miRNA-3162-3p were observed in *mice* lung cells for miRNA-943-3p, and the antagomir application of this molecule [57]. Among pediatric patients with an episode of asthma exacerbation, a decrease in miRNA-29c was noted in the blood, and its target point of action was the molecule B7-H3 (B7 protein homolog 3), the level of which was increased. It regulates Th2 and Th17 differentiation, cytokine synthesis, increased eosinophilic infiltration, and excessive mucus production. Infection of human monocytes obtained from laboratory cultures with a molecule that was a synthetic equivalent of miRNA-29c led to reduced expression of B7-H3 in an immunofluorescence assay in these cells [58,59]. Another complex research project was carried out on *mouse* lung cells and human bronchial epithelial cells (BEAS-2B). A complex pathway that could be a future therapeutic target for asthma is the miRNA-106b-5p/E2F1/SIX1. Increased levels of TGF-β1 were shown in asthmatics, which is responsible for the remodeling of the airways, transforming epithelial cells into myo/fibroblasts, subsequently leading to a decrease in the sensitivity to steroid therapy. Moreover, it intensifies the expression of SIX1 (sine oculis homeobox homolog 1), which mediates the change in the architecture of the bronchial tree. This study showed a decrease in the expression of miRNA-106b-5p, which directly results in an increase in SIX1 and E2F1 (E2F transcription factor 1), and TGF-β1 induces this process. Introducing the mimetic miRNA-106b-5p led to the inhibition of remodeling within the airways [60]. In airway smooth muscle cells obtained from asthmatics during bronchoscopy, miRNA-204-5p was found to play a similar role to the molecule described above. It inhibits TGF-β1-induced proliferation and extracellular matrix production within these cells by downregulating SIX1 [61]. A group of Chinese scientists exposed the epithelial cells of the *mouse* respiratory tract to house dust mites, causing an allergic inflammatory process. Subsequently, increased expression of miRNA-145-5p was observed, and the direct molecular target of this molecule was kinesin family member 3A (KIF3A), which was noted to be reduced in epithelial cells. This led to the increased production of IL-4, IL-5, and IL-13 derived from Th2 lymphocytes, which correlated with increased eosinophilic infiltration, mucus hypersecretion, and bronchial hyperresponsiveness. After using anti-miRNA-145-5p intranasally, all the above processes were inhibited [62]. Recently, it has been emphasized that exosomes may be involved in the pathogenesis of asthma [63,64]. This group of extracellular vesicles enables intercellular communication by carrying various molecules, including the same miRNA. The role of macrophage-derived exosomes was investigated using a *mouse* model of ovalbumin-induced asthma. It has been shown that after exposure of lung cells to their action, the fibrosis and inflammatory process are inhibited. The molecule responsible for this process is miRNA-370, which inactivates the MAPK/STAT (signal transducer and activator of transcription) pathway, which has key importance in the development of asthma [65]. In ASMC (airway smooth muscle cells) taken from adult subjects suffering from asthma during bronchoscopy, the increased production of the lncRNA (long non-coding RNA) NEAT1 (nuclear paraspeckle assembly transcript 1) was shown, which reduces the expression of miRNA-139 and is a signal for the phosphorylation of JAK3/STAT5 (Janus kinase 3/signal transducer and activator of transcription 5), leading to the synthesis of TNF-a, IL-6, IL-8, and IL-1b. The use of the miRNA-139 mimetic leads to the inhibition of the production of the above-mentioned pro-inflammatory substances and the development of the disease [66]. 

Th 17 (T helper 17 cells) are lymphocytes associated with severe asthma due to neutrophil infiltration and resistance to inhaled corticosteroids. It has been shown that the MBD2 protein (methyl-CpG-binding domain2) plays an important role in the differentiation of these cells, which allows recognition of methylated DNA and then regulation of its expression, making it a critical mediator of many epigenetic processes. Among the subjects diagnosed with severe asthma, it was shown that the level of miRNA-146a-3p was lower compared to the control group. The molecular target of this molecule was MBD2, the concentration of which was increased in asthmatics. The above relationships were simultaneously confirmed in *mouse* lung tissue. Inhalation of the mimic equivalent of miRNA-146a-3p results in a decrease in MBD2 and the number of Th17 lymphocytes involved in the inflammatory process, inhibition of mucus production, and airway hyperreactivity [67]. In the group of 68 pediatric patients with asthma, the reduction of miRNA-135b and the relationship of this molecule with the increase in CXCL12 (C-X-C motif chemokine ligand 12) were confirmed. It is responsible for increased inflammatory infiltration with the involvement of Th 17 lymphocytes and bronchial hyperreactivity. Anti-miRNA-135b and mimic miRNA-135b were applied by inhalation in *mice* in which ovalbumin supply induced the disease. In collected lung tissue samples from these *mice*, miRNA-135b was shown to lead to the inhibition of CXCL12 production and the infiltration of inflammatory cells [68]. RhoA (Ras Homolog Family Member A) is an essential protein associated with smooth muscle contraction in the airways, contributing to developing airway hyperresponsiveness. In human bronchial smooth muscle cells passaged under laboratory conditions, miRNA-140-3p was shown to be responsible for the expression of this protein. After using a mimetic of this molecule, there was a decrease in RhoA, followed by an inhibition of contraction [69]. Similar results were observed between the RhoA mRNA target gene and miRNA-140-3p in human bronchial smooth muscle cells [70]. miRNA-133a is also involved in regulating the RhoA pathway, and its increase inhibits airway constriction by limiting protein translation [71]. Another therapeutic target for asthma may be miRNA-375. In human airway epithelial cells, dexamethasone decreased this molecule and increased the synthesis of DUSP6 (dual specificity phosphatase 6), the target of miRNA-375 action. As shown, steroids, by affecting the miRNAs mentioned above (lowering the concentration), activate or inhibit several protein groups, contributing to the promotion of apoptosis within the cells of the respiratory tract, which is an important process that allows for maintaining normal function of the epithelial tissue [72]. Another critical pathway involved in the development of the disease is miRNA200a/miRNA200b/ORMDL3/ERK/MMP-9. Human respiratory epithelial cells were passaged in laboratory conditions and then mimetics and anti-miRNAs were introduced. It was observed that in samples where mimic miRNA-200a or miRNA-200b was used, there was a decrease in the expression of ORMDL3 (orosomucoid 1-like 3) and inhibition of ERK (extracellular signal-regulated kinase)/MMP-9 (matrix metalloproteinase-9) activation, and finally, a decrease in the amount of pro-inflammatory cytokines, including TNF-a, IL-4, IL-5, IL-13, and IL-1b. This is associated with stopping the inflammatory process and limiting the irreversible remodeling within the bronchial tree [73]. In vivo, after the application of dexamethasone to respiratory epithelial cells, the increased synthesis of GILZ (glucocorticoid-induced leucine zipper) was demonstrated, and decreased synthesis of miRNA-222-3p. GILZ is a transcription factor that inhibits the activation of the *MAPK* pathway, which has been shown to lead to the increased proliferation, migration, and differentiation of cells around the damaged site, playing a pivotal role in repairing the airway epithelium. The introduction of miRNA-222-3p led to a different therapeutic effect than that of steroids; a decrease in GILZ, promotion of the phosphorylation of signaling factors, and the activation of *MAPK*. The effect of these actions may be to promote repair of the damaged airway epithelium after using miRNA-222-3p [74]. Table 1 below summarizes the results of the studies presented above.

## 4. miRNAs and the Course of Viral Infections as a Factor of Asthma Exacerbation

An important aspect, especially among the pediatric population, are common acute viral infections, which are the leading cause of episodes of exacerbation of chronic inflammatory diseases in the respiratory tract, including asthma [75]. These exacerbations are associated with high morbidity and even mortality worldwide. It has been confirmed that miRNAs are induced during infection, which may play a key role in modulating the antiviral response. Therefore, it is very likely that miRNA changes originate from the infected epithelium and immune cells, which could lead to further dysregulation of airway inflammation [76,77]. In this chapter, we will focus on the potential role of miRNAs as novel molecules with therapeutic potential in acute viral and bacterial infections.

Rhinovirus (RV) is one of the most common pathogens and is the etiological factor of colds, bronchiolitis, and asthma exacerbations among children. The presence of RV in the respiratory tract secretions of infected *mice* led to the increased production of miRNA-122, which ultimately affected SOCS1 (suppressor of cytokine signaling 1), leading to a decrease in its expression and ultimately resulting in an increase in neutrophil infiltration in the airways. Experimental in vivo introduction of anti-miRNA-122 in animals reduced the inflammatory infiltration of the bronchoalveolar space, hypersensitivity of the airways, and triggered innate immune mechanisms, including IFN (interferon) production. A very interesting aspect is the fact that a higher level of miRNA-122 was found in the aspirate taken from the upper respiratory tract in infants hospitalized due to bronchiolitis, and the higher the expression of this molecule, the longer the need for oxygen therapy and the higher the rate of treatment failure [78]. In addition, early-life bronchiolitis is a strong risk factor for the development of asthma, and targeted antiviral therapy may prevent the future onset of the disease [79]. After infection of respiratory epithelial cells with RSV (respiratory syncytial virus) and the influenza virus in vitro, in addition to mimetic miRNA-24, -124a, and -744, a decrease in pathogen replication in the samples was shown by approximately 75%. The molecular target of these molecules is MK2 (MAPK-activated protein kinase 2) and its limited expression, which results in inhibition of the MAPK pathway activation and the spread of infection [80]. In another study, the potential therapeutic nature of anti-miRNA-146a was demonstrated in an infection caused by the influenza virus. The experiment was conducted in vivo (infecting *mice*) and in vitro on human respiratory epithelial cells. After contact with the pathogen, an increased concentration of miRNA-146a was unanimously noted in the tissues, inhibiting the production of type I IFN, which resulted in increased viral replication. After using an antagomir, there was a reduction in infection and damage to the *mouse* lung tissue [81]. In 2020, SARS-CoV-2 (severe acute respiratory syndrome coronavirus 2) became the cause of a worldwide pandemic and led to the death of many people. COVID-19 (coronavirus disease 2019) causes dysregulation of many inflammatory pathways (such as, for example, NF-kB and JAK/STAT), which are crucial in the occurrence of post-illness complications. It has been shown that the miRNA-9, miRNA-98, miRNA-223, and miRNA-214 molecules are responsible, at various stages, for inhibiting NF-kB and JAK/STAT, which reduces the inflammatory process and prevents the development of the disease [82]. Metapneumovirus (MPV) infection is another etiological factor of lower respiratory tract infections in infants and children. In epithelial cells, after their exposure to MPV, excessive synthesis of let-7f was noted, which led to inhibition of pathogen replication. A different effect was observed after infection of cells with RSV, which showed an increase in let-7f that induced viral replication. The opposite regulatory effect of this miRNA on hMPV and RSV replication suggests that it plays a different role depending on the etiological factor. Moreover, a different let-7f binding site has been demonstrated within the MPV and RSV genomes. Accordingly, using an antagomir or a mimetic of this molecule stops the pathogen replication [83]. In addition to viral infections, bacterial infections are another risk factor for asthma exacerbations. Widespread increasing resistance to antibiotics and impaired innate immunity in response to the use of steroids in the treatment of asthma is a common clinical problem. After infection with Haemophilus influenzae, the *mice* showed a decrease in the concentration of miRNA-328 in their lung macrophages. Using an antagomir of this molecule resulted in an increase in bactericidal activity by enhancing phagocytosis and the formation of reactive oxygen species. In addition, this study showed an interesting relationship where treatment with dexamethasone inhibited phagocytosis along with other inflammatory antibacterial pathways, while anti-miRNA-328 overcomes this effect. These results suggest that an antagomir could be combined with dexamethasone in treating chronic lung diseases to better control bacterial infections. This is particularly interesting for antibiotic-resistant bacterial strains where conventional therapies tend to fail and for patients receiving steroid therapy whose infections are difficult to control [84].

Currently, drugs based on the RNA structure are undergoing intensive preclinical and clinical trials. They can be divided into three classes: gene expression inhibitory (e.g., miRNA, siRNA (small interfering RNA), lncRNAs (long noncoding RNAs)), protein coding (e.g., mRNA), and protein targeting molecules (e.g., RNA aptamers) [85]. Compared to current drugs in use, RNA-based therapy offers additional advantages such as high selectivity and potency, the ability to provide personalized treatment, and rapid bioinformatics-based design [86]. The advantage of such preparations is their direct delivery to the target site of action by inhalation, minimizing potential systemic side effects. The main RNA therapeutics being investigated in obstructive airway diseases, including asthma, are siRNAs and, recently, miRNAs.

An example of their practical use was inhaling a specific inhibitor, miRNA-141-3p, which reduced mucus production and airway hyperresponsiveness in *mice* [87]. Despite the many benefits of the potential introduction of RNA-based inhalation drugs, there are also some limitations to their widespread use in medicine. The abundant presence of the nuclease in the body leads to the rapid breakdown of the drug, there is silencing of many genes off-target, and stimulation of the immune system can also occur. The hope for eliminating these negative phenomena is the chemical modification of RNA preparations [88].

The therapy for most cases of viral infections is based on symptomatic treatment, and in the case of available and specific antiviral drugs, it is focused on conservative proteins. However, this approach puts selective pressure on the pathogen and increases the likelihood of resistance to the drug. An alternative therapeutic strategy is to target host-encoded factors essential for the spread of infection, thereby minimizing the possibility of viral mutations that elude drug action. miRNAs can become these factor-influencing molecules [89].

## 5. Conclusions

The above review demonstrates the relationship of miRNAs with individual groups of drugs used in asthma, their involvement in pathways related to the disease pathogenesis, and their crucial role in developing viral infections. It is a rapidly evolving medical research field, as evidenced by the increasing number of publications. Determining the concentrations of miRNA molecules in the blood before, during, and after the end of therapy, as well as during episodes of asthma exacerbations, and their change in expression will make it possible to easily predict the expected therapeutic effect, assess the response to the treatment, as well as enabling a faster diagnosis before the symptoms of the disease process intensify. In addition, by combining reproducible methods of miRNA determination and their changing levels in the blood, the clinical characteristics of the patient, and available additional tests (e.g., spirometry), monitoring the effectiveness of therapy will be characterized by incredible accuracy. miRNAs can be used to classify and divide asthma cases into individual endotypes and/or phenotypes, which will allow optimal treatment selection, becoming the basis for introducing personalized medicine and a unique approach to each patient. Experimental translational studies conducted in the laboratory have confirmed the importance of miRNAs as potential molecules for future use in general clinical practice, although further refinement is still necessary. This unique opportunity presents itself for patients with severe asthma who do not respond to commonly used treatments, undoubtedly leading to an improved quality of life and significant economic implications by reducing the financial burden and costs associated with managing severe unresponsive asthma.

## Figures and Tables

**Table 1:** New potential directions for the therapeutic activities of miRNAs in asthma.

Study, Year	Experiment/Population	miRNA	Molecular Effect	The Biological Effect Obtained After Using Antagomir or Mirna
C. Huilong et al. [1], 2017	Induction of asthma by ovalbumin and analysis of cells in the bronchoalveolar lavage fluid and lung tissue/*MICE*	miRNA-155	Increased production of IL-4, 5, 13, and hypersensitivity of airway	Antagomir miRNA-155; inhibited the inflammatory process
J. Liu et al. [2], 2020	Induction of asthma by ovalbumin and analysis of cells in the bronchoalveolar lavage fluid/*MICE*	miRNA-3162-3p	Wnt/beta-catenin; increased production of the active form of beta-catenin, inflammatory response, and hypersensitivity of airway	Antagomir miRNA-3162-3p; anti-inflammatory effect such as GCs
J. Shen et al. [3], 2019	Induction of asthma by ovalbumin and analysis of cells in the bronchoalveolar lavage fluid and lung tissue/*MICE*	miRNA-943-3p	Wnt/SFRP-4; increased production of the active form of beta-catenin, inflammatory response, and fibrosis in the airway	Antagomir miRNA-943-3p; anti-inflammatory effect such as GCs
X. Zhang et al. [4], 2018	Analysis of blood sample/*CHILDREN* WITH ASTHMA EXCERBATIONS	miRNA-29c	B_7_-H_3_; increased eosinophilic infiltration, cytokine synthesis, mucus production, regulation of Th differentiation	Mimic miRNA-29c-inhibited the inflammatory process
S. Liu et al. [5], 2021	Induction of asthma by ovalbumin in *mice* and analysis of cells in the lung; human bronchial epithelial cells/*MICE*; *HUMAN*	miRNA-106b-5p	TGF-B1/SIX1/E2F1; initiated remodeling, fibrosis of the airways	Mimic miRNA-106b-5p; inhibited remodeling of the respiratory tract and increased sensitivity to the GCs
Z. Yang et al. [6], 2020	Smooth muscle cells collected during bronchoscopy/*HUMAN*	miRNA-204-5p	TGF-B1/SIX1; excessive smooth muscle cell proliferation and ECM production	Mimic miRNA-204-5p; suppressed the proliferation and ECM production of airway smooth muscle cells by directly regulating SIX1
T. Xiong et al. [7], 2019	Induction of allergic airways diseases by aeroallergen and analysis bronchial epithelial cells/*MICE*	miRNA-145-5p	KIF3-increased IL-4, 5, and 13 production, eosinophil infiltration, airway hyperresponsiveness	Antagomir miRNA-145; inhibited the inflammatory process
C. Li et al. [8], 2021	Induction of asthma by ovalbumin and isolated exosomes from macrophage/*MICE*	miRNA-370	MAPK/STAT; increased fibrosis and inflammatory process	Mimic miRNA-370; prevented airway remodeling and reduced inflammation
M. Zhu et al. [9], 2021	Smooth muscle cells collected during bronchoscopy/*HUMAN*	miRNA-139	lncRNA/JAK3/STAT5; increased production of TNF-a and IL-6, 8, and 1b	Mimic miRNA-139; reduced the production of cytokines
W. Duan et al. [10], 2023	Analysis of blood sample in severe asthma; *mouse* model of Th17 predominant neutrophilic, severe asthma/*HUMAN*; *MICE*	miRNA-146a-3p	MBD2; initialized severe asthma, increased Th17 lymphocytes, production of mucus, airway hyperresponsiveness	Mimic miRNA-146a-3p; suppressed production of Th17 lymphocytes and inflammatory process, development of severe asthma
Y. Liu et al. [11], 2022	Analysis of blood sample; induction of asthma by ovalbumin in *mice* and analysis bronchial epithelial cells/*CHILDREN*; *MICE*	miRNA-135b	CXCL-12; increased inflammatory process with Th17, airway hyperresponsiveness	Mimic miRNA-135b; decreased CXCL-12 production and inflammatory infiltration
Y. Chiba et al. [12], 2021	Bronchial smooth muscle cell/*HUMAN*	miRNA-140-3p	RhoA; increased the strength contraction of smooth muscle	Mimic miRNA-140-3p; inhibited contraction of smooth muscle
X. Duan et al. [13], 2020	Bronchial epithelial cells/*HUMAN*	miRNA-200a/200b	ORMDL3/ERK/MMP-9; increased production of TNF-a, IL-4, IL-5, IL-13, IL-1b, and the inflammatory process	Mimic miRNA-200a/200b; inhibited the inflammatory process
N. He et al. [14], 2020	Bronchial epithelial cells treated by dexamethasone/*HUMAN*	miRNA-222-3p	GILZ; inhibited proliferation, migration, and cell differentiation around the damaged epithelium	Mimic 222-3p; promoted the repair of damaged epithelium

NOTE: Fourth column—increase in the concentration of all molecules except for SFRP4 and KIF-3. Abbreviations: IL—Interleukins; GCs—glucocorticosteroids; SFRP—4-secreted frizzled-related proteins 4; s-H_3_-B7 homolog 3 protein; TGF-β1—transforming growth factor β1, SIX1—sine oculis homeobox homolog 1; KIF3A—kinesin family member 3A; E2F1—E2F transcription factor 1; ECM—extracellular matrix; lncRNA—nuclear paraspeckle assembly transcript 1; JAK3/STAT5—janus kinase 3/signal transducer and activator of transcription 5; MBD-2—methyl-CpG-binding domain2; CXCL-12—C-X-C motif chemokine ligand 12; RhoA—Ras Homolog Family Member A; ORMDL3—orosomucoid 1-like 3; ERK—extracellular signal regulated kinase; MMP-9—matrix metalloproteinase-9; GILZ—glucocorticoid-induced leucine zipper.

## Data Availability

No new data were created or analyzed in this study. Data sharing is not applicable to this article.
